# Mitral and tricuspid valve repair in a patient with eosinophilic granulomatosis with polyangiitis

**DOI:** 10.1093/jscr/rjag838

**Published:** 2026-09-20

**Authors:** Anna Dąbrowska, Maria Skorupa, Mateusz Kozioł, Janusz Konstanty-Kalandyk

**Affiliations:** Medical College, Jagiellonian University, 12 St. Anne's Street, 31-008 Krakow, Poland; Medical College, Jagiellonian University, 12 St. Anne's Street, 31-008 Krakow, Poland; Medical College, Jagiellonian University, 12 St. Anne's Street, 31-008 Krakow, Poland; Department of Cardiovascular Surgery and Transplantology, John Paul 2nd Hospital, Jagiellonian University, 80 Prądnicka Street, 31-202 Krakow, Poland; Medical College, Jagiellonian University, 12 St. Anne's Street, 31-008 Krakow, Poland; Department of Cardiovascular Surgery and Transplantology, John Paul 2nd Hospital, Jagiellonian University, 80 Prądnicka Street, 31-202 Krakow, Poland

**Keywords:** eosinophilic granulomatosis with polyangiitis, mitral valve replacement, tricuspid valve regurgitation

## Abstract

Eosinophilic granulomatosis with polyangiitis (EGPA) is an autoimmune disease which most serious complication is cardiac involvement. In our knowledge in the available literature there wasn’t any description of surgery of both mitral and tricuspid valves in connection with a complication after EGPA. A 49-year-old patient with previously diagnosed EGPA was admitted to the Cardiosurgery Department due to mitral and tricuspid valve regurgitation. The patient was qualified to the surgery—the ATS 27 mm valve was implanted in the mitral position and the tricuspid valve repair was conducted using Physio 32 mm ring via a transseptal approach. Postoperative period was uneventful and the patient was discharged in good condition. Cardiac involvement is the main cause of death in patients with EGPA. Appropriate diagnosis, treatment and perioperative management could be challenging, require knowledge and experience in management of this condition.

## Introduction

Eosinophilic granulomatosis with polyangiitis (EGPA), previously called as Churg–Strauss syndrome, is characterized by a triad of hypereosinophilia, asthma, and necrotizing vasculitis of small to medium arteries in people with a history of atopy [1]. Etiology of this disease is still unknown although it has been consistently found that 30%–40% of affected patients have antineutrophil cytoplasmic antibodies (ANCA) [2]. The valve insufficiency in patients with EGPA is a quite rare finding and mostly involve mitral and tricuspid valve [3].

## Case report

A 49-year-old patient with postcapillary pulmonary hypertension, EGPA, bronchial asthma, arterial hypertension, dyslipidemia, chronic sinusitis, tracheal stenosis after tracheostomy, multifocal mononeuropathy, after NSTEMI 6 years ago and after pericarditis and myocarditis in the process of EGPA was admitted to the hospital for further diagnostics of mitral and tricuspid valve regurgitation. The patient recently reported dyspnea on exertion and decreased exertion tolerance. During physical examination, the systolic murmur was detected in the V intercostal space radiating to the left armpit.

**Figure 1:**
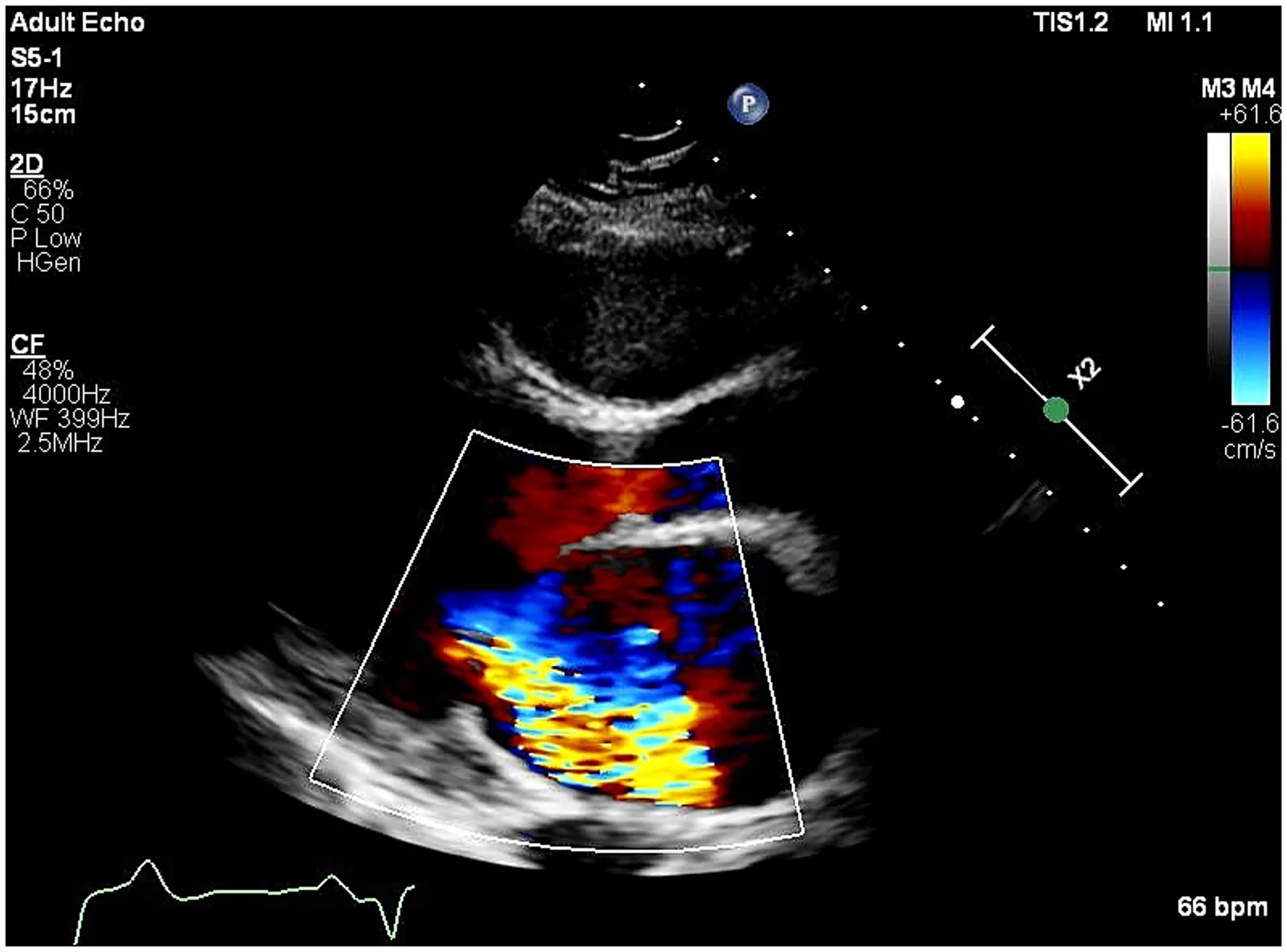
Mitral valve regurgitation.

**Figure 2:**
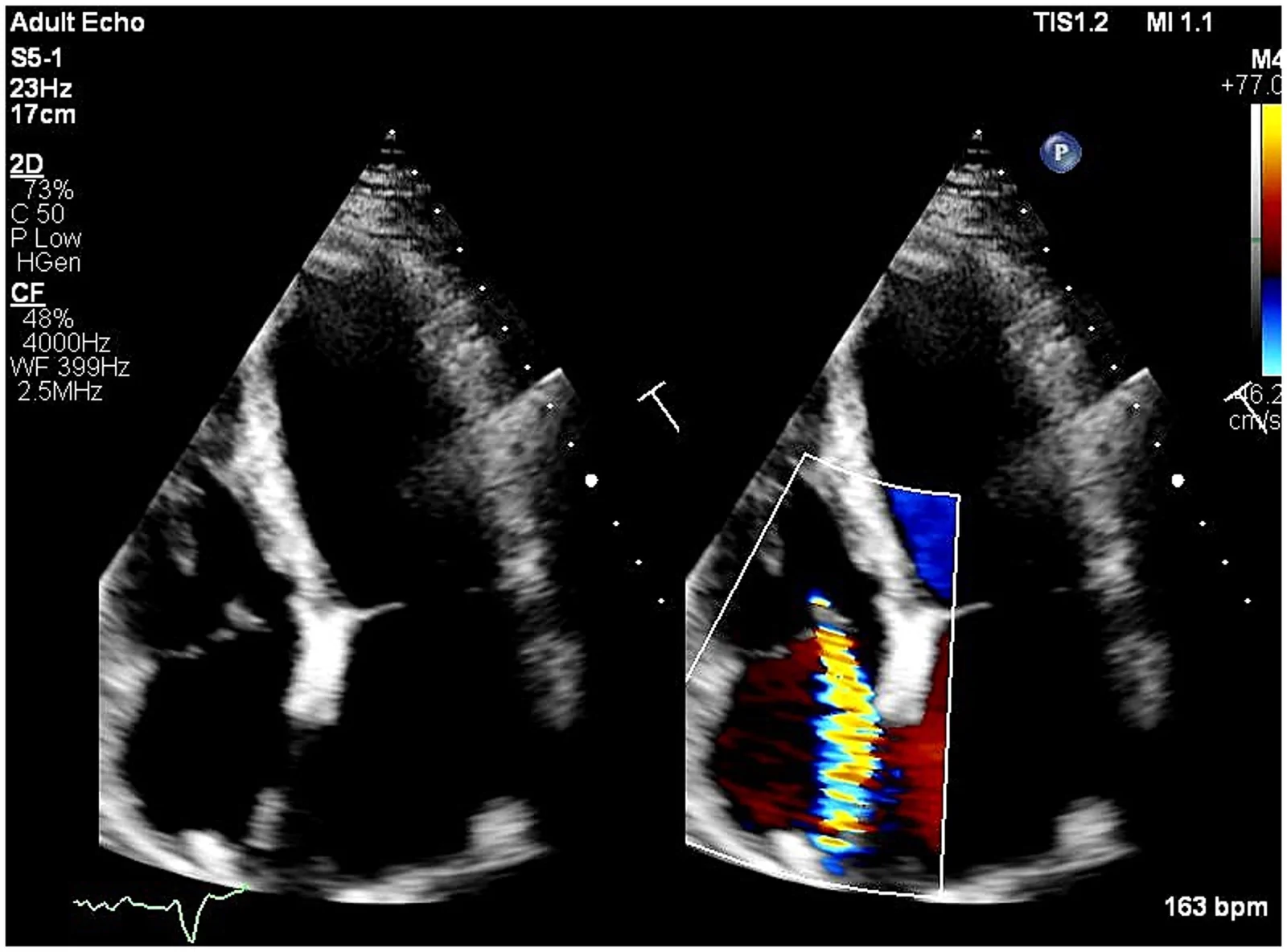
Tricuspid valve regurgitation.

Echocardiography showed enlargement all four heart chambers, preserved global left ventricle contractility, left ventricle ejection fraction 60%, severe mitral valve regurgitation (Vena Contracta Width: 6 mm, Effective Regurgitant Orifice: 0,34 cm2) (Fig. 1), moderate tricuspid valve regurgitation (Right Ventricular Systolic Pressure: 53 + 3 mmHg) (Fig. 2), mild aortic and pulmonary regurgitation. Coronarography hasn’t shown any stenosis in the coronary vessels. Our patient was consulted by the multidisciplinary team ‘Heart Team’ and was qualified to mitral and tricuspid valvuloplasty in 3 months. Patient was discharged from the hospital. A month before surgery, doctors performed tests measuring progression of EGPA. The remission of EGPA was found and discontinuation of azathioprine was proposed in the perioperative period.

Patient was admitted to the hospital for surgery as planned. After thoracotomy, multiple pericardial adhesions were found. The ATS 27 mm valve was implanted in the mitral position via a transseptal approach. The tricuspid valve repair was conducted using Physio 32 mm ring. In the postoperative period, the increase of inflammatory parameters was detected and empiric antibiotic therapy was provided. Thanks to the treatment, an improvement in the clinical condition and a decrease in inflammatory parameters were achieved. Due to anemia, 2 units of blood cell concentrate were transfused without complications. There were no complications during the postoperative period and wound healing. The patient was discharged from the hospital in a good general condition, cardiovascular and respiratory efficiency.

## Discussion

EGPA is an autoinflammatory disease, a type of necrotizing vasculitis of small- to medium-sized arteries which co-occur with respiratory tract involvement. 2022 American College of Rheumatology/European Alliance of Associations for Rheumatology Classification Criteria for EGPA allows to diagnose EGPA in patients with small- or medium-vessel vasculitis, who collected at least 6 points in following categories: obstructive airway disease (+3 points), nasal polyps (+3 points), mononeuritis multiplex (+1 point), blood eosinophil count ≥1x109/liter (+5 points), extravascular eosinophilic-predominant inflammation on biopsy (+2 points), positive test for cytoplasmic antineutrophil cytoplasmic antibodies (cANCA) or antiproteinase 3 (anti-PR3) antibodies (−3 points) and hematuria (−1 point) [4]. Our patient presented with asthma, chronic sinusitis, mononeuritis multiplex (confirmed in electromyogram), blood eosinophil count 4270/ul, eosinophilic infiltration of bone marrow, negative test for cANCA, anti-PR3 antibodies and no hematuria received 14 points, what with the presence of vasculitis is enough to making a diagnosis. The most significant example of vasculitis was in the coronary arteries, which led to non-STEMI myocardial infarction. Coronarography preformed after occurring the ischemia didn’t reveal any hemodynamically significant atherosclerotic plaque.

Cardiac involvement is the main cause of death in patients with EGPA. In particular, endocarditis has a high mortality rate [5]. Neumann *et al*. report that EGPA may have the following heart abnormalities: coronary heart disease, right or left bundle branch block, ST and T-wave abnormalities, ventricular arrhythmias, pericardial effusions, mitral, aortic or tricuspid valve insufficiencies, reduced left ventricular ejection fraction, pulmonary hypertension, presence of thrombotic structures in the ventricular chambers, elevated troponin I level or Löffler endocarditis with the formation of fibrotic tissue observable in biopsy. The cited studies also suggest that loss of ANCA is associated with cardiac involvement in patients with EGPA. In the presented case, no ANCA was present, what is supported by study’s outcome. The article reveals also that cardiac function could be improved with immunosuppressive treatment.

To our knowledge, the patient hadn’t performed neither heart biopsy nor histopathological examination of replaced mitral valve. However, MRI imaging revealed changes typical for EGPA. Baccouche *et al*. [6] showed that MRI imaging of EGPA is associated with the presence of eosinophilic infiltration and hemorrhagic necrosis in cardiac involvement of these disease. Therefore, we assume that EGPA was the main cause of valve insufficiency in our patient.

Histological findings in patients with cardiac involvement include acute eosinophilic endomyocarditis and typical histologic signs of Löffler endocarditis with the formation of fibrotic tissue. However, due to invasive character the indications for heart muscle biopsy must be carefully considered.

Perioperative management in patients with EGPA requires careful administration of anesthetic drugs due to upper airway involvement [7]. Propofol that was used in our patient, has been reported to cause less bronchoconstriction during anesthetic induction than other anesthetic agents [8] and therefore was the best choice for anesthetic induction. Also, opioids are thought to be safe in asthmatic patients, but it is important that they are injected slowly to avoid thorax rigidity what can mimic bronchospasm. Opioid’s suppression of the cough reflex is the benefit helpful in management of EGPA patients. We used sufentanil from this group of drugs to achieve deep anesthesia. It is also important to choose the appropriate muscle relaxant agent. Jooste *et al.* [9]. reported that medications with greater affinity to type 2 than type 3 muscarinic acetylcholine receptors like gallamin, pipecuronium, rapacuronium could cause bronchoconstriction in sensitive patients. It is therefore recommended to use drugs like vecuronium, rocuronium, cisatracurium or pancuronium. From this group of drugs, we administered pancuronium to the patient. At the end of anesthesia, it is important to avoid reversal of muscle relaxation, because neostigmine and physostigmine could cause bronchial hyper-reactivity and increase secretion in patient’s airways.
